# Correlation of parent-reported physical health-related quality-of-life with quantitatively measured physical function of former critically ill children

**DOI:** 10.1186/s41687-026-01064-7

**Published:** 2026-04-15

**Authors:** Nazlı Umman Serin, Jan Gunst, Fabian Güiza, Karolijn Dulfer, Sascha Verbruggen, Koen Joosten, Greet Van den Berghe, Ilse Vanhorebeek

**Affiliations:** 1 Clinical Division and Laboratory of Intensive Care Medicine, Department of Cellular and Molecular Medicine, KU Leuven, Leuven, Belgium; 2 Division of Pediatric Intensive Care Unit, Department of Neonatal and Pediatric ICU, Erasmus Medical Center, Sophia Children’s Hospital, Rotterdam, The Netherlands

**Keywords:** Critical illness, Pediatric intensive care unit, Physical functional performance, Child, Health-related quality-of-life

## Abstract

**Background:**

Many children admitted to a pediatric intensive-care-unit (PICU) show impaired physical function years later. Testing physical function clinically is time-consuming and expensive, hampering routine follow-up. We investigated whether subjective parent-reported physical health-related quality-of-life correlates with and may replace objectively measured physical function of former PICU-patients.

**Methodology:**

This secondary analysis of the PEPaNIC-RCT included 517 former PICU-patients with physical function tests and parent-reported health-related quality-of-life 4 years later. Parents scored physical function, bodily pain, general health and physical role functioning with questionnaires. Physical function tests included handgrip-strength, timed up-and-go-test, 6-minute-walk-test, and accelerometry. Questionnaire-items and test-scores were correlated with Pearson/Spearman correlation.

**Results:**

Parent-reported physical function correlated moderately with daily time in sedentary state (ρ=-0.300, *p* < 0.0001), daily sedentary bouts (*R*=-0.330, *p* < 0.0001) and daily steps/hour monitored (*R* = 0.303, *p* < 0.0001), and weakly with handgrip-strength, physical activity energy expenditure, time in certain activity-intensity and daily steps (ρ/*R* = 0.185–0.289, *p* ≤ 0.019). Parent-reported general health (ρ/*R*=-0.228 to 0.296, *p* ≤ 0.033) and physical role functioning (ρ/*R*=-0.269 to 0.240, *p* ≤ 0.038) correlated weakly with roughly the same measured physical functions. Parent-reported bodily pain correlated weakly with daily sedentary bouts (*R*=-0.143, *p* = 0.024) and daily steps/hour monitored (*R* = 0.145, *p* = 0.022). Correlations among physical function test scores were mostly weak.

**Conclusions:**

Several aspects of subjective parent-reported physical health-related quality-of-life correlated with former PICU-patients’ performance on physical function tests. The mostly weak strength of the correlations suggest that interrogated physical function cannot simply replace clinical testing. Nevertheless, when resources for clinical testing are lacking, interrogating physical function could still be valuable to get an idea about long-term physical functioning.

**Supplementary Information:**

The online version contains supplementary material available at 10.1186/s41687-026-01064-7.

## Introduction

Many children who required admission to a pediatric intensive care unit (PICU) for extensive vital organ support are confronted with a wide range of long-term health and developmental problems, together labeled as post-intensive care syndrome in children [1–3]. In addition to neurocognition, mental health and behavior, also their physical function is often affected. Conclusions on physical function after pediatric critical illness have mostly been drawn from parent-reported physical components in health-related quality-of-life questionnaires or scales based on observer impressions [4–6]. Interrogating a child’s health-related quality-of-life or observing physical function is relatively easy but, although this provides important information, has the disadvantage of yielding data on an individual’s perception which hence remain subjective. Recently, we quantitatively assessed several physical function measures in former critically ill children 4-years after PICU-admission, which showed less handgrip-strength, worse functional dynamic balance, worse functional exercise capacity and less overall daily physical activity as compared with healthy children [7]. Whereas these measurements have the advantage of being objective, they have the disadvantage of requiring a large investment of time and money as opposed to the use of questionnaires or scales.

We aimed to investigate whether aspects of subjective parent-reported health-related quality-of-life correlate with quantitatively measured physical function and whether such correlation would be strong enough to allow replacement of quantitative measurements with interrogated physical health-related quality-of-life after pediatric critical illness.

## Methods

### Study design and participants

This study reports a secondary analysis of the ‘Early versus Late Parenteral Nutrition in the PICU randomized controlled trial (PEPaNIC RCT, registered at ClinicalTrials.gov NCT01536275 on February 16, 2012) and its 4-year follow-up study [8–10]. This large multicenter RCT was conducted in PICUs in Belgium, the Netherlands and Canada between June 2012-July 2015. The study was designed to study the impact on short- and long-term outcome of early versus late initiation of supplemental parenteral nutrition (PN) when enteral nutrition was insufficient in critically ill children. All surviving former PICU-patients were eligible for a follow-up study four years after PICU-admission, with detailed investigation of their physical, neurocognitive and emotional/behavioral development, which showed impairments in all domains as compared with healthy children (2016–2019) [7, 10]. The participating sites’ institutional review boards approved the original and follow-up studies (Ethical Committee Research UZ/KU Leuven: ML8052; Medische Ethische Toetsingscommissie Erasmus MC: NL49708.078), which were performed in accordance with the 1964 Declaration of Helsinki and its amendments. Written informed consent was acquired from parents, legal guardians and/or the child if 18 years or older.

**Fig. 1:**
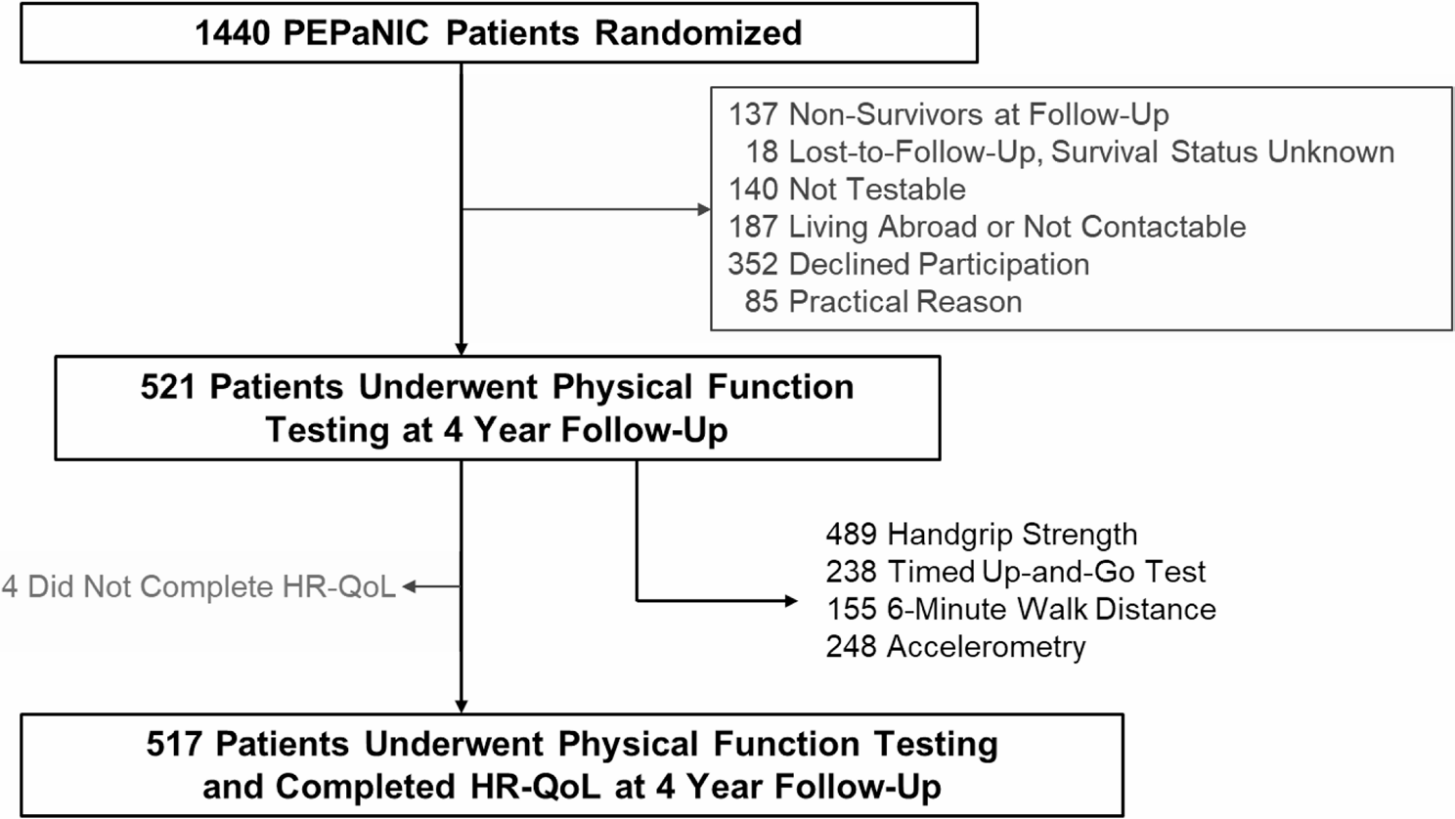
Consort diagram of study participants. Abbreviations: HR-QoL: health-related quality-of-life

For this study we selected all former PEPaNIC patients who underwent quantitative testing of physical function (only performed in Leuven and Rotterdam) and for whom parents/caregivers reported health-related quality-of-life four years after PICU-admission (*n* = 517, Fig. 1).

### Parent-reported physical health-related quality-of-life

Health-related quality-of-life represents the impact of health on physical, emotional, and social well-being and provides insights into the impact of health problems on the daily life of a person. The parents of children aged 4–18 years were asked to complete the Child Health Questionnaire Parent Form 50 (CHQ-PF50) to evaluate their children’s health-related quality-of-life, similarly as described for the 2-year follow-up of the same cohort [11]. The CHQ-PF50 has 50 questions, which are grouped into 11 multi-item scales and 4 single-item scales. For this study, we selected the scales on physical health-related quality-of-life, including physical functioning, bodily pain, general health and physical role functioning, each with scores ranging from 0 to 100. *Physical functioning* represents the ability to do basic physical activities such as eating, sleeping, grasping and playing. A lower score indicates significant limitations in performing these activities due to health-related problems, whereas a higher score suggests that the child has no or minor health-related restrictions to carry out these tasks. *Bodily pain* assesses the frequency and severity of bodily pain felt by the child, where a lower score indicates frequent, intense pain, while a higher score indicates no or minimal pain or discomfort for the child’s daily functioning. *General health* reflects the parent’s general awareness of the child’s health. Perceptions of poor health and potential decline are indicated by lower scores, while perceptions of good health and stability are indicated by higher scores. *Physical role functioning* evaluates the child’s ability to participate in school-related activities and social interactions with peers in relation to physical health. A lower score indicates significant limitations in these areas, while a higher score indicates no or minimal restrictions in social engagement or in school due to health-related problems.

### Quantitatively measured physical function

Physical functional capacity was assessed via measurement of handgrip-strength, via performance on the timed up-and-go test and the 6-minute walk test, and via an accelerometer-based evaluation of overall physical activity in daily life, as previously described in detail [7]. Children aged 4 years or older are able to complete these tests.

*Handgrip-strength* for the dominant and non-dominant hand was measured with a Jamar Plus + digital hand dynamometer (Patterson Medical Ltd., Nottinghamshire, UK). Average scores of three measurements were converted from kilograms to percentage predicted for age and sex. The “*timed up-and-go*” test evaluates functional dynamic balance. The test involved timing of the duration required to rise from a chair (both feet flat on the floor, knees at 90-degree angle), to walk 3 m, turn around a cone, come back to the chair, and sit down again. The fastest recorded time from three attempts was retained. The *6-minute walk test* was used to assess functional exercise capacity. Participants were asked to walk (not run) along a straight flat corridor as quickly as they could for six minutes. The total distance covered in six minutes was recorded. *Overall daily life physical activity* was comprehensively assessed via monitoring with an ActiGraph accelerometer (model wGT3X-BT, ActiGraph Corporation, Pensacola, FL). Participants were asked to wear the device at their right hip for seven consecutive days during waking hours and remove it only for sleep and water-based activities (e.g. showering, bathing or swimming). To ensure the accuracy of the data, parents or study participants were asked to meticulously log the times the monitor was worn or removed. Data were obtained on estimated total energy expenditure (kcal/kg/day and kcal/kg/hour monitored), physical activity-related energy expenditure (expressed as metabolic equivalent of task (MET), which compares working metabolic rate to resting metabolic rate), and activity intensity (categorized by time spent in sedentary, light (< 3.0 METs), moderate (3.0-5.9 METs), vigorous (6.0-8.9 METs), or very vigorous (≥ 9.0 METs) activity, expressed as percentage of total monitored time. The number of Freedson bouts (periods of at least 10 min of moderate-to-vigorous activity) was also measured, as were the number of sedentary bouts (periods of ≥ 10 min in a sedentary state) and the total number of steps taken (measured per day and per hour monitored). All data were averaged across the recorded days. Data for a participant were only considered for evaluation if activity had been registered for at least 5 out of 7 days (of which at least one weekend day) for at least 8 h/day.

### Statistical analyses

Patient characteristics are reported as medians with interquartile ranges or as numbers with percentages. Missing data on health-related quality-of-life were imputed by chained equations [10, 12]. All data available for each individual were used. Bias and instability of the imputation model was minimized by only including outcomes with no more than 30% missing data and the number of imputation models was set at 31 to avoid the loss of statistical power. Percentage of imputed data was 16% for physical function, bodily pain and general health, and 17% for physical role functioning. Correlations between quantitative physical function measures and items of physical health-related quality-of-life were performed with Pearson’s or Spearman’s correlation. Pearson’s correlation was used if the residuals for a linear fit showed a normal distribution, as assessed with use of the Shapiro-Wilk test (if necessary after square root transformation of the raw data), otherwise Spearman’s correlation was used. We similarly assessed correlations among the quantitative physical function measures. Correlation coefficients < 0.3 indicate weak correlations, those 0.3-<0.6 indicate moderate correlations, and those ≥ 0.6 indicate strong correlations.

All statistical analyses were done with JMP ©Pro17.0.0 (SAS Institute, Inc., Cary, NC). A *p* ≤ 0.05 was considered statistically significant. Since the outcomes examined are not independent and this is an exploratory study, no adjustments for multiple comparisons were made.

## Results

### Participants

The combination of parent/caregiver-reported health-related quality-of-life and any data on physical function at 4-year follow-up was available for 517 former PICU-patients included in the PEPaNIC trial (Fig. 1). Baseline characteristics of all 517 former PICU-patients are shown in Table 1 and of those who completed a specific functional test in Online Resource Table A1. Scores for physical health-related quality-of-life and results of physical function tests are shown in Table 2.

**Table 1:** Baseline characteristics of the former PICU patients

Characteristic	Former PICU patients
Age at 4-year follow-up (year), median (IQR)	5.08 (4.45–8.47)
Male sex, no (%)	300 (58.0)
Known non-European origin, no (%)	78 (15.1)
Known non-Caucasian race, no (%) ^a^	36 (7.0)
Known not exclusive Dutch or English language, no (%)	110 (21.3)
Socioeconomic status ^b, c^	
Parenteral educational level 1, no (%)	66 (12.8)
Parenteral educational level 2, no (%)	226 (43.7)
Parenteral educational level 3, no (%)	147 (28.4)
Parenteral educational level unknown, no (%)	78 (15.1)
Parenteral occupational level 1, no (%)	53 (10.3)
Parenteral occupational level 2, no (%)	140 (27.1)
Parenteral occupational level 3, no (%)	137 (26.5)
Parenteral occupational level 4, no (%)	83 (16.1)
Parenteral occupational level unknown, no (%)	104 (20.1)
STRONGkids risk level – medium / high, no (%) ^d^	470 (90.9) / 47 (9.1)
PeLOD score first 24 h in PICU, median (IQR) ^e^	21 (12–32)
PIM3 score / PIM3 probability of death (%), median (IQR) ^f, g^	-3.8 (-4.4 to -2.8) / 2.1 (1.2–5.8)
Diagnostic category, no (%)	
Surgical - Abdominal, no (%)	42 (8.1)
Surgical - Burns, no (%)	3 (0.6)
Surgical - Cardiac, no (%)	248 (48.0)
Surgical – Neurosurgery-traumatic brain injury, no (%)	40 (7.7)
Surgical - Thoracic, no (%)	30 (7.7)
Surgical - Transplantation, no (%)	7 (1.4)
Surgical - Orthopedic surgery—trauma, no (%)	13 (2.5)
Surgical - Other, no (%)	20 (3.9)
Medical - Cardiac, no (%)	16 (3.1)
Medical - Gastrointestinal–hepatic, no (%)	1 (0.2)
Medical - Oncologic–hematologic, no (%)	6 (1.2)
Medical - Neurologic, no (%)	26 (5.0)
Medical - Renal, no (%)	0 (0.0)
Medical - Respiratory, no (%)	43 (8.3)
Medical - Other, no (%)	22 (4.3)
History of malignancy, no (%)	24 (4.6)
History of diabetes, no (%)	0 (0.0)
Syndrome, no (%) ^h^	39 (7.5)
Known parental smoking between birth and PICU admission, no (%)	134 (25.9)

^a^ Participants were classified according to race and geographical origin by the investigators. ^b^ The education level is the average of the paternal and maternal educational level, and calculated based on the 3-point scale subdivisions as made by the Algemene Directie Statistiek (Belgium) and the Centraal Bureau voor de Statistiek (Netherlands). Low (1), middle (2), and high (3) educational level. ^c^ The occupation level is the average of the paternal and maternal occupation level, which is calculated based on the International ISCO System 4-point scale for professions. ^d^ STRONGkids scores range from 0 to 5, with a score of 0 indicating a low risk of malnutrition, a score of 1 to 3 indicating a medium risk, and a score of 4 to 5 indicating a high risk. ^e^ PeLOD scores range from 0 to 71, with higher scores indicating more severe illness. ^f^ Higher PIM3 scores indicate a higher risk of mortality. ^g^ PIM3 probability of death, ranging from 0% to 100%, with higher percentages indicating a higher probability of death in PICU. ^h^ A prerandomization syndrome or illness a priori defined as affecting or possibly affecting neurocognitive development

Abbreviations: IQR: interquartile range, PeLOD: pediatric logistic organ dysfunction score, PICU: pediatric intensive care unit, PIM3: pediatric index of mortality 3 score. STRONGkids: Screening Tool for Risk on Nutritional Status and Growth

**Table 2:** Physical health-related quality-of-life and measured physical functions of the former PICU patients

Outcome	Median (IQR)
**Physical health-related quality-of-life** (*n* = 517)	
Physical function ^a^	100 (72–100)
Bodily pain ^a^	80 (61–100)
General health ^a^	52 (44–68)
Physical role functioning ^a^	100 (71–100)
**Quantitative measurements**	
Handgrip-strength (% of predicted) (*n* = 486)	
Average force dominant hand	94.0 (77.5–117.0)
Average force non-dominant hand	91.9 (74–111.0)
Timed up-and-go test (s) (*n* = 237)	5.5 (4.9–6.3)
6-minute walk test	
Distance walked (m) (*n* = 155)	485 (420–587)
Actigraph ^b^ (*n* = 247)	
Daily time monitored (h)	12.0 (11.4–12.7)
Total energy expenditure	
kcal/kg/day	107.6 (86.6-157.3)
kcal/kg/hour monitored	9.3 (7.2–12.6)
Physical activity energy expenditure	
Metabolic Equivalent of Task	2.1 (1.7–2.4)
Daily time spent in a type of activity, % ^c^	
Sedentary	47.4 (41.5–52.8)
Light activity	46.1 (21.6–52.2)
Moderate activity	6.0 (3.9–18.9)
Vigorous activity	0.9 (0.4-2.0)
Very vigorous activity	0.1 (0.0-0.6)
Moderate to vigorous activity	8.0 (4.8–22.5)
Number of Freedson bouts	0.8 (0.2–1.5)
Number of sedentary bouts	6.0 (4.4–8.9)
Number of steps walked	
Steps/day	8838 (7299–10308)
Steps/day/hour monitored	741 (601–867)

Data are expressed as median and interquartile range. ^a^ Scores range from 0 to 100. ^b^ Data averaged over the registered days. ^c^ Expressed as the percentage of worn/monitored time

### Correlation between parent-reported physical health-related quality-of-life and quantitatively measured physical function of former PICU-patients

Table 3 shows the correlations between different aspects of physical health-related quality-of-life and individual quantitatively measured physical functions. Parent-reported *physical function* showed moderate negative correlations with percentage of daily time spent in sedentary activity (ρ=-0.300, *p* < 0.0001) and daily number of sedentary bouts (*R*=-0.330, *p* < 0.0001) and moderate positive correlation with the number of steps walked/day/hour monitored (*R* = 0.303, *p* < 0.0001). Parent reported-physical function further showed weak positive correlations with handgrip-strength; estimated physical activity energy expenditure; percentage of daily time spent in light activity, vigorous activity and very vigorous activity; and number of steps walked/day. *Bodily pain* showed a weak negative correlation with number of sedentary bouts and a weak positive correlation with number of steps walked/day. *General health* showed weak positive correlations with handgrip-strength; estimated total energy expenditure; percentage of daily time spent in light, vigorous and very vigorous activity; number of Freedson bouts and number of steps walked, and weak negative correlations with percentage of daily time spent in sedentary activity and number of sedentary bouts. *Physical role functioning* showed weak positive correlations with handgrip-strength; estimated physical activity energy expenditure; percentage of daily time spent in vigorous and very vigorous activity; and number of steps walked; and weak negative correlations with percentage of daily time spent in sedentary activity and number of sedentary bouts.

**Table 3:** Association between physical health-related quality-of-life and quantitatively measured physical functions of former PICU patients

Outcome	Physical function	Bodily pain	General health	Role functioning - physical
Handgrip-strength (% of predicted)				
Average force dominant hand	**ρ = 0.185**, ***p*** **< 0.0001**^a^	ρ = 0.035, *p* = 0.44	**ρ = 0.184**, ***p*** **< 0.0001**^a^	**ρ = 0.094**, ***p*** **= 0.038**^a^
Average force non-dominant hand	**ρ = 0.197**, ***p*** **< 0.0001**^a^	ρ = 0.049, *p* = 0.28	**ρ = 0.158**, ***p*** **= 0.0005**^a^	**ρ = 0.103**, ***p*** **= 0.024**^a^
Timed up-and-go test (s)	ρ = -0.075, *p* = 0.25	ρ = -0.048, *p* = 0.46	ρ = -0.070, *p* = 0.28	ρ = 0.124, *p* = 0.057
6-minute walk test				
Distance walked (m)	*R* = -0.095, *p* = 0.24	ρ = -0.108, *p* = 0.18	ρ = 0.040, *p* = 0.62	*R* = -0.094, *p* = 0.24
Actigraph				
Total energy expenditure				
kcal/kg/day	ρ = 0.006, *p* = 0.92	ρ = 0.003, *p* = 0.96	**ρ = 0.154**, ***p*** **= 0.015**^a^	ρ = -0.007, *p* = 0.91
kcal/kg/hour monitored	ρ = 0.043, *p* = 0.50	ρ = 0.019, *p* = 0.76	**ρ = 0.159**, ***p*** **= 0.012**^a^	ρ = 0.021, *p* = 0.74
Physical activity energy expenditure				
Metabolic Equivalent of Task	**ρ = 0.289**, ***p*** **= 0.0043**^a^	ρ = 0.059, *p* = 0.56	ρ = 0.109, *p* = 0.29	**ρ = 0.237**, ***p*** **= 0.020**^a^
Daily time spent in a type of activity, %				
Sedentary	**ρ = -0.300**, ***p*** **< 0.0001**^b^	ρ = -0.100, *p* = 0.14	**ρ = -0.228**, ***p*** **= 0.0007**^a^	**ρ = -0.218**, ***p*** **= 0.0012**^a^
Light activity	**ρ = 0.187**, ***p*** **= 0.0032**^a^	ρ = 0.062, *p* = 0.33	**ρ = 0.166**, ***p*** **= 0.0089**^a^	ρ = 0.114, *p* = 0.075
Moderate activity	ρ = -0.068, *p* = 0.28	ρ = -0.032, *p* = 0.61	ρ = 0.045, *p* = 0.48	ρ = -0.076, *p* = 0.23
Vigorous activity	**ρ = 0.189**, ***p*** **= 0.0029**^a^	ρ = 0.055, *p* = 0.38	**ρ = 0.296**, ***p*** **< 0.0001**^a^	**ρ = 0.171**, ***p*** **= 0.0071**^a^
Very vigorous activity	**ρ = 0.237**, ***p*** **= 0.019**^a^	ρ = 0.016, *p* = 0.87	**ρ = 0.217**, ***p*** **= 0.033**^a^	**ρ = 0.213**, ***p*** **= 0.036**^a^
Moderate to vigorous activity	ρ = -0.039, *p* = 0.54	ρ = -0.034, *p* = 0.59	ρ = 0.074, *p* = 0.24	ρ = -0.045, *p* = 0.47
Number of Freedson bouts	ρ = 0.123, *p* = 0.053	ρ = 0.037, *p* = 0.56	**ρ = 0.185**, ***p*** **= 0.0035**^a^	ρ = 0.110, *p* = 0.084
Number of sedentary bouts	***R*** **= -0.330**, ***p*** **< 0.0001**^b^	***R*** **= -0.143**, ***p*** **= 0.024**^a^	**ρ = -0.160**, ***p*** **= 0.011**^a^	**ρ = -0.269**, ***p*** **< 0.0001**^a^
Number of steps walked				
Steps/day	***R*** **= 0.225**, ***p*** **= 0.0004**^a^	*R* = 0.109, *p* = 0.086	***R*** **= 0.208**, ***p*** **= 0.0010**^a^	***R*** **= 0.195**, ***p*** **= 0.0021**^a^
Steps/day/hour monitored	***R*** **= 0.303**, ***p*** **< 0.0001**^b^	***R*** **= 0.145**, ***p*** **= 0.022**^a^	***R*** **= 0.207**, ***p*** **= 0.0011**^a^	***R*** **= 0.240**, ***p*** **= 0.0001**^a^

Correlations with *p* < 0.05 are indicated in bold. ^a^ Weak correlations (R or ρ < 0.3), ^b^ Moderate correlations (R or ρ 0.3 - <0.6)

Like the correlations between physical health-related quality-of-life and measured physical function, correlations among quantitatively measured physical functions were also mostly weak (Online Resource Table A2).

## Discussion

We documented several significant correlations between aspects of parent-reported physical health-related quality-of-life and quantitatively measured physical outcomes of former PICU-patients 4 years after PICU-admission. These correlations were mostly weak and mainly found for parent-reported physical function, general health and physical role functioning, whereas hardly any correlations were found for bodily pain. The mostly weak strength of these correlations was roughly comparable to that of the correlations among the quantitatively measured physical functions themselves.

Parent-reported physical function showed a moderate negative correlation with the daily time spent in sedentary activity and number of sedentary bouts, as well as a moderate positive correlation with the number of steps walked/day/hour monitored. Parent-reported physical function further showed weak positive correlations with handgrip-strength, physical activity energy expenditure and daily time spent in light, vigorous and very vigorous activity. This is plausible as the more parents judge that health-related problems limit their child in basic physical activities, the less active their child would be and vice versa. Likewise, interrogated general health and physical role functioning showed weak correlations with measured handgrip-strength, total or physical activity energy expenditure, time spent in a certain intensity of activity and number of steps walked. Thus, perceptions of poor health and limitations to participate in school-related activities and social interactions with peers associated with worse measured physical function. In contrast, bodily pain only correlated with number of sedentary bouts and number of steps/day walked. This may suggest that discomfort by pain perception may in general be less broadly limiting in former critically ill children. In contrast, in a convenience sample of British children, an association was found between pain as deduced from the Pediatric Pain questionnaire and physical activity, though only for time spent in vigorous and not moderate physical activity [13].

Finding correlations between subjective and objective measures of physical function is reassuring. However, the overall rather weak strength of these correlations suggests, at first sight, that parent-reported physical health-related quality-of-life reveals some information on physical function of former PICU patients but cannot replace objective comprehensive clinical testing of physical function in long-term follow-up. Both types of measures have their own value and give complementary information. Objective quantitative measures of physical function are important for assessing specific abilities and evaluating progression or efficacy of an intervention. Physical health-related quality-of-life provides a broader, holistic view of how someone’s physical health is perceived and how a disease or disability affects daily life. Interestingly, however, correlations among quantitatively measured aspects of physical function were also mostly weak. In this perspective, the correlations between subjective and objectively quantified outcomes may be even more striking. As such, when resources for comprehensive clinical testing of physical functioning are lacking, our findings may support the use of interrogating physical health with quality-of-life questionnaires in clinical follow-up to get an idea about physical functioning.

We did not find any other studies on association of interrogated health-related quality-of-life with objectively measured physical function in (former) critically ill children. However, such association studies have been performed in children in other contexts. These studies were mostly small and yielded divergent results, where some found correlations that agreed with our findings, whereas others did not find any correlation between measured and interrogated physical function. Two studies in children with cerebral palsy made use of the physical component score of the CHQ-PF50 questionnaire, the questionnaire that we used. These studies also found (moderate) associations with several accelerometer data, including physical activity energy expenditure, percentage of time spent in sedentary or moderate-to-vigorous physical activity and/or activity counts [14, 15]. A study in children with multimorbidity or physical illness showed a moderate correlation of physical health-related quality-of-life rated by the parents with the KIDSCREEN-27 questionnaire with moderate-to-vigorous physical activity as recorded with an accelerometer [16]. Most other studies made use of the Pediatric Quality-of-life Inventory (PedsQL) questionnaire. In children with cerebral palsy, scores on the parent- or self-reported physical domain, and the total scores, of this questionnaire showed moderate correlations with physical activity energy expenditure, percentage of time spent in moderate-to-vigorous physical activity and activity counts [14]. The total score of this questionnaire also showed moderate correlation with sedentary behavior and moderate-to-vigorous physical activity time of Hispanic children in the US [17]. No associations were found of the PedsQL physical function and/or total score with accelerometer-quantified physical activity measures (light/moderate/vigorous activity, moderate-to-vigorous physical activity, sedentary behavior, or daily step count) in children with idiopathic toe walking, in children two months after a concussion, or in general populations of children in England or Malaysia [18–21]. Also, no association was observed between general health status as assessed by the HSD010 questionnaire and accelerometer-quantified physical activity in a general US adolescent population [22].

Of note, none of the physical health-related quality-of-life aspects correlated with 6-minute walk distance of former PICU-patients. The former PICU-patients showed more pronounced exertional oxygen desaturation in the 6-minute walk test as compared with healthy children [7]. Whether this exertional desaturation may explain the lack of correlation between physical health-related quality-of-life and 6-minute walk distance remains unclear. The lack of correlation with 6-minute walk distance agrees with studies in children with multiple sclerosis [23] and healthy children [24], that respectively used the PedsQL and Child Health Questionnaire-28 to evaluate physical health-related quality-of-life. In contrast, a positive correlation has been documented between the parent-proxy- or self-reported total PedsQL score and 6-minute walk distance of children with cancer [25].

Our study has some limitations that should be discussed. First, the studied cohort comprised only former PICU-patients who came to the hospital for this follow-up study and who were not too disabled to be physically tested. This may have biased the analyses towards more physically active children. Second, as data were obtained 4 years after PICU-admission, former PICU-patients were minimally 4 years old at assessment, hence not allowing extrapolation to younger ages. Third, physical health-related quality-of-life was deduced from parent-reports and not self-reports as the majority of participants were too young to comprehend the questionnaire items. Fourth, the Actigraph accelerometer could not assess water-based activities. Finally, the correlations between subjective parent-reported health-related quality-of-life and objective quantitative measures of physical activity were studied in a cross-sectional design, which does not allow causal inference. However, it was not the objective of this study to establish such causality.

## Conclusions

Several aspects of subjective parent-reported physical health-related quality-of-life correlated with former PICU patients’ performance on physical function tests, though mostly only weakly. This suggests that comprehensive clinical testing cannot simply be replaced with interrogated physical function. Nevertheless, although captured information is not completely equivalent, when resources for comprehensive clinical testing of physical functioning are lacking, interrogating physical health with quality-of-life questionnaires could be valuable in clinical follow-up to get a rough idea about physical functioning of these vulnerable patients.

## Supplementary Information

Below is the link to the electronic supplementary material.

Supplementary Material 1

## Data Availability

The datasets generated and/or analyzed during the current study are not publicly available due to ethical constraints. Data sets and other documents may be shared on reasonable request under the format of future collaborative projects that further elaborate on this specific research topic. Proposals for collaborative projects must be directed to the corresponding author and will be considered for approval. The data set required to address the research question will then only be shared after signing of a data access agreement. Data are located in controlled access data storage at KU Leuven.

## Abbreviations

CHQ-PF50: Child Health Questionnaire Parent Form 50
MET: Metabolic equivalent of task
PEDSQL: Pediatric quality-of-life inventory
PEPaNIC: Pediatric early vs late parenteral nutrition in intensive care unit
PICU: Pediatric intensive care unit
PN: Parenteral nutrition
RCT: Randomized controlled trial

## References

[1] Manning JC, Pinto NP, Rennick JE, Colville G, Curley MAQ (2018) Conceptualizing Post Intensive Care Syndrome in Children-The PICS-p Framework. Pediatr Crit Care Med 19:298–300. 10.1097/PCC.0000000000001476 29406379 10.1097/PCC.0000000000001476

[2] Woodruff AG, Choong K (2021) Long-term outcomes and the post-intensive care syndrome in critically ill children: a North American perspective. Child (Basel) 8. 10.3390/children8040254 10.3390/children8040254 PMC8064072 33805106

[3] Long DA, Fink EL (2021) Transitions from short to long-term outcomes in pediatric critical care: considerations for clinical practice. Transl Pediatr 10:2858–2874. 10.21037/tp-21-61 34765507 10.21037/tp-21-61 PMC8578758

[4] Hordijk JA, Verbruggen SC, Buysse CM, Utens EM, Joosten KF, Dulfer K (2022) Neurocognitive functioning and health-related quality of life of children after pediatric intensive care admission: a systematic review. Qual Life Res 31:2601–2614. 10.1007/s11136-022-03124-z 35357629 10.1007/s11136-022-03124-z PMC9356943

[5] Procter C, Morrow B, Pienaar G, Shelton M, Argent A (2021) Outcomes following admission to paediatric intensive care: A systematic review. J Paediatr Child Health 57:328–358. 10.1111/jpc.15381 33577142 10.1111/jpc.15381

[6] Pollack MM, Holubkov R, Funai T, Clark A, Moler F, Shanley T et al (2014) Relationship between the functional status scale and the pediatric overall performance category and pediatric cerebral performance category scales. JAMA Pediatr 168:671–676. 10.1001/jamapediatrics.2013.5316 24862461 10.1001/jamapediatrics.2013.5316 PMC4589215

[7] Vanhorebeek I, Jacobs A, Mebis L, Dulfer K, Eveleens R, Van Cleemput H et al (2022) Impact of critical illness and withholding of early parenteral nutrition in the pediatric intensive care unit on long-term physical performance of children: a 4-year follow-up of the PEPaNIC randomized controlled trial. Crit Care 26:133. 10.1186/s13054-022-04010-3 35549984 10.1186/s13054-022-04010-3 PMC9097055

[8] Fivez T, Kerklaan D, Mesotten D, Verbruggen S, Wouters PJ, Vanhorebeek I et al (2016) Early versus Late Parenteral Nutrition in Critically Ill Children. N Engl J Med 374:1111–1122. 10.1056/NEJMoa1514762 26975590 10.1056/NEJMoa1514762

[9] Fivez T, Kerklaan D, Verbruggen S, Vanhorebeek I, Verstraete S, Tibboel D et al (2015) Impact of withholding early parenteral nutrition completing enteral nutrition in pediatric critically ill patients (PEPaNIC trial): study protocol for a randomized controlled trial. Trials 16:202. 10.1186/s13063-015-0728-8 25927936 10.1186/s13063-015-0728-8 PMC4422419

[10] Jacobs A, Dulfer K, Eveleens RD, Hordijk J, Van Cleemput H, Verlinden I et al (2020) Long-term developmental effect of withholding parenteral nutrition in paediatric intensive care units: a 4-year follow-up of the PEPaNIC randomised controlled trial. Lancet Child Adolesc Health 4:503–514. 10.1016/S2352-4642(20)30104-8 32562632 10.1016/S2352-4642(20)30104-8

[11] Hordijk J, Verbruggen S, Vanhorebeek I, Guiza F, Wouters P, Van den Berghe G et al (2020) Health-related quality of life of children and their parents 2 years after critical illness: pre-planned follow-up of the PEPaNIC international, randomized, controlled trial. Crit Care 24:347. 10.1186/s13054-020-03059-2 32546247 10.1186/s13054-020-03059-2 PMC7296688

[12] Wulff JJ (2017) Multiple imputation by chained equations in praxis:guidelines and review. Electron J Bus Res Methods 15:41–56

[13] de Aguiar Greca JP, Korff T, Ryan J (2021) Associations Between Children’s Physical Activity, Pain and Injuries. Percept Mot Skills 128:1959–1974. 10.1177/00315125211028455 34187240 10.1177/00315125211028455 PMC8414821

[14] Lee J, Suk MH, Yoo S, Kwon JY (2022) Physical activity energy expenditure predicts quality of life in ambulatory school-age children with cerebral palsy. J Clin Med 11. 10.3390/jcm11123362 10.3390/jcm11123362 PMC9225112 35743433

[15] Yoon MJ, Choi H, Kim JS, Lim SH, Yoo YJ, Hong BY (2022) Physical activity, quality of life and parenting stress in children with cerebral palsy. Pediatr Int 64:e15295. 10.1111/ped.15295 10.1111/ped.15295 36112040

[16] Bedard C, King-Dowling S, Obeid J, Timmons BW, Ferro MA (2022) Correlates of moderate-to-vigorous physical activity in children with physical illness and physical-mental multimorbidity. Health Educ Behav. 2022;49(5):10901981221100697. 10.1177/10901981221100697 10.1177/10901981221100697 PMC9465499 35695286

[17] Gu X, Zhang T, Chen S, Keller MJ, Zhang X (2020) School-based sedentary behavior, physical activity, and health-related outcomes among hispanic children in the United States: a cross-sectional study. Int J Environ Res Public Health 17. 10.3390/ijerph17041197 10.3390/ijerph17041197 PMC7068440 32069928

[18] Caserta A, Reedman S, Morgan P, Williams CM (2022) Physical activity and quality of life in children with idiopathic toe walking: a cross sectional study. BMC Pediatr 22:544. 10.1186/s12887-022-03583-w 36100938 10.1186/s12887-022-03583-w PMC9472422

[19] Eagle SR, Zynda AJ, Sandulli L, Hickey RW, Kegel NE, Nelson L et al (2025) The Role of Body Mass Index on Physical Activity, Symptoms, and Related Outcomes Following Pediatric Concussion. J Pediatr 277:114386. 10.1016/j.jpeds.2024.114386 39489284 10.1016/j.jpeds.2024.114386

[20] Tinner L, Kipping R, White J, Jago R, Metcalfe C, Hollingworth W (2019) Cross-sectional analysis of physical activity in 2-4-year-olds in England with paediatric quality of life and family expenditure on physical activity. BMC Public Health 19:846. 10.1186/s12889-019-7129-y 31253117 10.1186/s12889-019-7129-y PMC6599301

[21] Wafa SW, Shahril MR, Ahmad AB, Zainuddin LR, Ismail KF, Aung MM et al (2016) Association between physical activity and health-related quality of life in children: a cross-sectional study. Health Qual Life Outcomes 14:71. 10.1186/s12955-016-0474-y 27146199 10.1186/s12955-016-0474-y PMC4857334

[22] Gao Y, Zhang Q, Wang Y, Gao Y, Xu Y (2024) Association between physical activity based on wearables and self-reported health status among adolescents: NHANES 2011–2014. BMC Public Health 24:2876. 10.1186/s12889-024-20363-6 39425121 10.1186/s12889-024-20363-6 PMC11490019

[23] Zenginler Yazgan Y, Vural P, Ormen R, Akinci B, Tarakci E, Guler S et al (2022) Six-Minute Walk Performance and Related Factors in Pediatric-Onset Multiple Sclerosis. J Child Neurol 37:351–358. 10.1177/08830738211072701 35317699 10.1177/08830738211072701

[24] Baldwin JN, McKay MJ, Hiller CE, Moloney N, Nightingale EJ, Burns J (2017) Relationship between physical performance and self-reported function in healthy individuals across the lifespan. Musculoskelet Sci Pract 30:10–17. 10.1016/j.msksp.2017.05.001 28494261 10.1016/j.msksp.2017.05.001

[25] Keating R, Curry S, Hussey J (2023) Cardiorespiratory fitness and health-related quality of life in survivors of childhood central nervous system tumours. Support Care Cancer 31:395. 10.1007/s00520-023-07854-9 37318588 10.1007/s00520-023-07854-9 PMC10272264

