# Supplementary material for: Correlation of parent-reported physical health-related quality-of-life with quantitatively measured physical function of former critically ill children
Source: J Patient Rep Outcomes. 2026 Apr 15;10:86. doi: 10.1186/s41687-026-01064-7 (PMC13201699; doi:10.1186/s41687-026-01064-7)
Supplement: Supplementary file 1 — Supplementary Material 1 [file 41687_2026_1064_MOESM1_ESM.docx]

**QUALITY OF LIFE RESEARCH**

**SUPPLEMENTARY INFORMATION**

**Correlation of parent-reported physical health-related quality-of-life with quantitatively measured physical function of former critically ill children**

Nazlı Umman Serin,^1^ Jan Gunst,^1^ Fabian Güiza,^1^ Karolijn Dulfer,^2^ Sascha C. Verbruggen,^2^ Koen F. Joosten,^2^ Greet Van den Berghe,^1^ Ilse Vanhorebeek^1^

^1^ Clinical Division and Laboratory of Intensive Care Medicine, Department of Cellular and Molecular Medicine, KU Leuven, Leuven, Belgium; ^2^ Division of Pediatric Intensive Care Unit, Department of Neonatal and Pediatric ICU, Erasmus Medical Center, Sophia Children’s Hospital, Rotterdam, The Netherlands.

Corresponding author: Ilse Vanhorebeek, Laboratory of Intensive Care Medicine, KU Leuven, Herestraat 49, B-3000 Leuven, Belgium. Email: ilse.vanhorebeek@kuleuven.be; ORCID 0000-0002-5261-5192

Other ORCIDs: Nazlı Umman Serin: 0000-0001-8215-8909; Jan Gunst: 0000-0003-2470-6393; Fabian Güiza: 0000-0001-7026-0957; Karolijn Dulfer: 0000-0002-0158-6539; Sascha C. Verbruggen: 0000-0003-4866-9865; Koen F. Joosten: 0000-0002-0504-2475; Greet Van den Berghe: 0000-0002-5320-1362

**TABLE OF CONTENTS**

**Table A1.** Baseline characteristics of former PICU patients who completed individual physical function tests

**Table A2.** Associations among quantitatively measured physical outcomes of former PICU patients

**Table A1.** **Baseline characteristics of former PICU patients who completed individual physical function tests**

|  | **Former PICU patients with physical function testing** | | | |
| --- | --- | --- | --- | --- |
| **Characteristic** | **TUG** | **HGF** | **6MWD** | **Actigraph** |
| n | 283 | 486 | 155 | 247 |
| Age at 4-year follow-up (year), median (IQR) | 5.08 (4.44-8.66) | 5.13 (4.47-8.65) | 5.90 (4.52-10.66) | 5.04 (4.45-7.69) |
| Male sex, no (%) | 140 (59.1) | 285 (58.6) | 88 (56.8) | 147 (59.5) |
| Known non-European origin, no (%) | 34 (14.3) | 72 (14.8) | 17 (11.0) | 33 (13.4) |
| Known non-Caucasian race, no (%) ^a^ | 21 (8.9) | 34 (7.0) | 14 (9.0) | 15 (6.1) |
| Known not exclusive Dutch or English language, no (%) | 37 (15.6) | 99 (20.4) | 19 (12.3) | 45 (18.2) |
| Socioeconomic status ^b,c^ |  |  |  |  |
| Parenteral educational level 1, no (%) | 30 (12.7) | 64 (13.2) | 21 (13.5) | 25 (10.1) |
| Parenteral educational level 2, no (%) | 108 (45.6) | 208 (42.8) | 74 (47.7) | 112 (45.3) |
| Parenteral educational level 3, no (%) | 71 (30.0) | 138 (28.4) | 43 (27.7) | 82 (33.2) |
| Parenteral educational level unknown, no (%) | 28 (11.8) | 76 (15.6) | 17 (11.0) | 28 (11.3) |
| Parenteral occupational level 1, no (%) | 17 (7.1) | 45 (9.3) | 12 (7.7) | 28 (11.3) |
| Parenteral occupational level 2, no (%) | 63 (26.6) | 132 (27.2) | 46 (29.7) | 66 (26.7) |
| Parenteral occupational level 3, no (%) | 69 (29.1) | 127 (26.1) | 42 (27.1) | 79 (32.0) |
| Parenteral occupational level 4, no (%) | 44 (18.6) | 80 (16.5) | 22 (14.2) | 42 (17.0) |
| Parenteral occupational level unknown, no (%) | 44 (18.6) | 102 (21.0) | 33 (21.3) | 32 (13.0) |
| STRONGkids risk level – medium / high, no (%) ^d^ | 214 (90.3) / 23 (9.7) | 441 (90.7) / 45 (9.3) | 136 (87.7) / 19 (12.3) | 227 (91.9) / 20 (8.1) |
| PeLOD score first 24 h in PICU, median (IQR) ^e^ | 21 (12-31) | 21 (12-31) | 21 (11-31) | 22 (21-32) |
| PIM3 score ^f^ | -3.9 (-4.4 to -2.8) | -3.9 (-4.4 to -2.8) | -3.7 (-4.5 to -2.6) | -3.9 (-4.4 to -2.9) |
| PIM3 probability of death (%), median (IQR) ^g^ | 2.1 (1.2-5.5) | 2.1 (1.2-5.8) | 2.4 (1.1-7.1) | 2.1 (1.2-5.3) |
| Diagnostic category, no (%) |  |  |  |  |
| Surgical - Abdominal, no (%) | 19 (8.0) | 40 (8.2) | 16 (10.3) | 16 (6.5) |
| Surgical - Burns, no (%) | 1 (0.4) | 3 (0.6) | 1 (0.6) | 0 (0.0) |
| Surgical - Cardiac, no (%) | 105 (44.3) | 229 (47.1) | 59 (38.1) | 143 (57.9) |
| Surgical – Neurosurgery-traumatic brain injury, no (%) | 16 (6.8) | 39 (8.0) | 14 (9.0) | 14 (5.7) |
| Surgical - Thoracic, no (%) | 12 (5.1) | 27 (5.6) | 7 (4.5) | 13 (5.3) |
| Surgical - Transplantation, no (%) | 2 (0.8) | 7 (1.4) | 2 (1.3) | 2 0.8) |
| Surgical - Orthopedic surgery—trauma, no (%) | 4 (1.7) | 12 (2.5) | 2 (1.3) | 6 (2.4) |
| Surgical - Other, no (%) | 8 (3.4) | 19 (3.9) | 8 (5.2) | 11 (4.5) |
| Medical - Cardiac, no (%) | 10 (4.2) | 14 (2.9) | 7 (4.5) | 9 (3.6) |
| Medical - Gastrointestinal–hepatic, no (%) | 0 (0.0) | 1 (0.2) | 0 (0.0) | 1(0.4) |
| Medical - Oncologic–hematologic, no (%) | 4 (1.7) | 6 (1.2) | 4 (2.6) | 1 (0.4) |
| Medical - Neurologic, no (%) | 15 (6.3) | 25 (5.1) | 8 (5.2) | 9 (3.6) |
| Medical - Renal, no (%) | 0 (0.0) | 0 (0.0) | 0 (0.0) | 0 (0.0) |
| Medical - Respiratory, no (%) | 29 (12.2) | 42 (8.6) | 20 (12.9) | 11 (4.5) |
| Medical - Other, no (%) | 12 (5.1) | 22 (4.5) | 7 (4.5) | 11 (4.5) |
| History of malignancy, no (%) | 14 (5.9) | 22 (4.5) | 12 (7.7) | 8 (3.2) |
| History of diabetes, no (%) | 0 (0.0) | 0 (0.0) | 0 (0.0) | 0 (0.0) |
| Syndrome, no (%) ^h^ | 10 (4.2) | 32 (6.6) | 5 (3.2) | 15 (6.1) |
| Known parental smoking between birth and PICU admission, no (%) | 65 (27.4) | 129 (26.5) | 48 (31.0) | 50 (20.1) |

^a^ Participants were classified according to race and geographical origin by the investigators. ^b^ The education level is the average of the paternal and maternal educational level, and calculated based on the 3-point scale subdivisions as made by the Algemene Directie Statistiek (Belgium) and the Centraal Bureau voor de Statistiek (Netherlands). Low (1), middle (2), and high (3) educational level. ^c^ The occupation level is the average of the paternal and maternal occupation level, which is calculated based on the International ISCO System 4-point scale for professions. ^d^ STRONGkids scores range from 0 to 5, with a score of 0 indicating a low risk of malnutrition, a score of 1 to 3 indicating a medium risk, and a score of 4 to 5 indicating a high risk. ^e^ PeLOD scores range from 0 to 71, with higher scores indicating more severe illness. ^f^ Higher PIM3 scores indicate a higher risk of mortality. ^g^ PIM3 probability of death, ranging from 0% to 100%, with higher percentages indicating a higher probability of death in PICU. ^h^ A prerandomization syndrome or illness a priori defined as affecting or possibly affecting neurocognitive development.

Abbreviations: 6MWD: 6-minute walk distance, HGF: handgrip force, IQR: interquartile range, PeLOD: pediatric logistic organ dysfunction score, PICU: pediatric intensive care unit, PIM3: pediatric index of mortality 3 score. STRONGkids: Screening Tool for Risk on Nutritional Status and Growth, TUG: timed up-and-go test

**Table A2: Associations among quantitatively measured physical functions of former PICU patients**

|  | **Handgrip-strength**  **dominant hand** | **Handgrip-strength**  **non-dominant hand** | **Timed up-and-go test (s)** | **6-minute walk distance (m)** |
| --- | --- | --- | --- | --- |
| **Outcome** |  |  |  |  |
| Handgrip-strength (% of predicted) |  |  |  |  |
| Average force dominant hand | - | **-** | **-** | **-** |
| Average force non-dominant hand | **ρ = 0.814, p < 0.0001** ^c^ | - | - | - |
| Timed up-and-go test (s) | ρ = -0.070, p = 0.28 | ρ = -0.043, p = 0.51 | - | - |
| 6-minute walk test |  |  |  |  |
| Distance walked (m) | **R = -0.159, p = 0.050** ^a^ | ρ = -0.141, p = 0.082 | **ρ = -0.633, p < 0.0001** ^c^ | - |
| Actigraph |  |  |  |  |
| Total energy expenditure |  |  |  |  |
| kcal/kg/day | ρ = -0.069, p = 0.31 | ρ = -0.061, p = 0.37 | **ρ = -0.398, p < 0.0001** ^b^ | **R = 0.511, p < 0.0001** ^b^ |
| kcal/kg/hour monitored | ρ = -0.044, p = 0.51 | ρ = -0.035, p = 0.61 | **ρ = -0.414, p < 0.0001** ^b^ | **R = 0.495, p < 0.0001** ^b^ |
| Physical activity energy expenditure |  |  |  |  |
| Metabolic Equivalent of Task | **R = 0.245, p = 0.024** ^a^ | **R = 0.299, p = 0.0051** ^a^ | R = 0.058, p = 0.69 | **ρ = -0.370, p = 0.034** ^b^ |
| Daily time spent in a type of activity, % |  |  |  |  |
| Sedentary | **ρ = -0.152, p = 0.037** ^a^ | **ρ = -0.154, p = 0.034** ^a^ | ρ = -0.088, p = 0.34 | **R = 0.269, p = 0.044** ^a^ |
| Light activity | **ρ = 0.134, p = 0.050** ^a^ | ρ = 0.098, p = 0.15 | **ρ = 0.237, p = 0.0065** ^a^ | R = 0.221, p = 0.066 |
| Moderate activity | **ρ = -0.159, p = 0.019** ^a^ | **ρ = -0.173, p = 0.010** ^a^ | **ρ = -0.507, p < 0.0001** ^b^ | **R= 0.636, p < 0.0001** ^c^ |
| Vigorous activity | **ρ = 0.152, p = 0.025** ^a^ | **ρ = 0.163, p = 0.016** ^a^ | **ρ = -0.183, p = 0.036** ^a^ | **ρ = 0.286, p = 0.016** ^a^ |
| Very vigorous activity | **R = 0.309, p = 0.0040** ^b^ | **R = 0.343, p = 0.0012** ^b^ | R = 0.007, p = 0.96 | R= 0.012, p = 0.94 |
| Moderate to vigorous activity | ρ = -0.116, p = 0.089 | ρ = -0.122, p = 0.074 | **ρ = -0.509, p < 0.0001** ^b^ | **R = 0.659, p < 0.0001** ^c^ |
| Number of Freedson bouts | **ρ = 0.174, p = 0.010** ^a^ | **ρ = 0.171, p = 0.011** ^a^ | **ρ = -0.317, p = 0.0002** ^b^ | **R = 0.285, p = 0.016** ^a^ |
| Number of sedentary bouts | **R = -0.172, p = 0.011** ^a^ | **ρ = -0.161, p = 0.018** ^a^ | **ρ = -0.186, p = 0.033** ^a^ | **R = 0.522, p < 0.0001** ^b^ |
| Number of steps walked |  |  |  |  |
| Steps/day | **R = 0.138, p = 0.042** ^a^ | **ρ = 0.135, p = 0.047** ^a^ | **ρ = -0.192, p = 0.028** ^a^ | R = 0.033, p = 0.78 |
| Steps/day/hour monitored | **R = 0.172, p = 0.011** ^a^ | **ρ = 0.166, p = 0.014** ^a^ | ρ = -0.163, p = 0.062 | R = 0.042, p = 0.73 |

Correlations with p < 0.05 are indicated in bold. ^a^ Weak correlations (R or r < 0.3), ^b^ Moderate correlations (R or r 0.3 - < 0.6), ^c^ Strong correlations (R or r ≥ 0.6).
